# Focused ultrasound-induced blood–brain barrier opening to enhance interleukin-12 delivery for brain tumor immunotherapy: a preclinical feasibility study

**DOI:** 10.1186/s12967-015-0451-y

**Published:** 2015-03-17

**Authors:** Pin-Yuan Chen, Han-Yi Hsieh, Chiung-Yin Huang, Chun-Yen Lin, Kuo-Chen Wei, Hao-Li Liu

**Affiliations:** Department of Neurosurgery, Chang-Gung Memorial Hospital, Taoyuan, Linkou Taiwan; Department of Electrical Engineering, Chang-Gung University, Taoyuan, Taiwan; Department of Hepatogastroenterology, Chang-Gung Memorial Hospital, Taoyuan, Taiwan; Healthy Aging Research Center, Chang-Gung University, Taoyuan, Taiwan; School of Medicine, Chang-Gung University, Taoyuan, Taiwan; Medical Imaging Research Center, Institute for Radiological Research, Chang Gung University and Chang Gung Memorial Hospital at Linkou, Taoyuan, Taiwan

**Keywords:** Focused ultrasound, Blood–brain barrier, IL-12, Immune therapy

## Abstract

**Background:**

Interleukin-12 (IL-12) has long been considered to be effective in triggering an anticancer immune response, however, the dosage has been limited by potential systemic immunotoxicity. Since focused ultrasound (FUS) has been confirmed to temporally and locally open the blood–brain barrier (BBB), the purpose of this study was to elucidate the possibility of combining FUS-induced BBB opening with IL-12 delivery to enhance the anticancer immunological response for glioma treatment.

**Methods:**

FUS energy combined with microbubble administration was delivered transcranially to open BBB, and C-6 glioma rats were used in this study. The efficacy in inducing BBB opening and the corresponding immunological response were primarily evaluated in normal animals. The anticancer immune-triggering chemokine, IL-12, was intraperitoneally administered during the treatment phase to evaluate the effect of immunological response on tumor progression. Glioma animals were sub-grouped to evaluate the effect of the immune response in suppressing glioma when IL-12 was combined with FUS-induced BBB opening. We performed flow cytometry to verify consequent immune cell population changes of peripheral/tissue lymphocytes as well as macrophages from the animals. Brain sections of sacrificed animals were also used for histological and immunohistochemical analysis. IL-12 level among experimental groups were measured via ELISA analysis. We also analyzed survival and followed tumor progression *in vivo* via T2-weighted magnetic resonance imaging.

**Results:**

FUS-induced BBB opening had no obvious effect on the T lymphocytes population in normal animals, either in the brain or systemically. Yet, it triggered mild changes in the tumor-infiltrating lymphocyte (TIL) population, particularly in numbers of CD3+CD8+ cytotoxic T lymphocytes (CTLs) in the tumor region. IL-12 administration triggered a profound increase in all TIL populations, including CD3+CD4+ T helper cells (Th), CTL, and CD4+CD25+ regulatory T cells (Treg), but combined FUS-BBB opening with IL-12 administration produced the most significant IL-12 increase, CTL increase and CTL/Treg ratio increase, thus contributing to the most significant suppression of tumor progression and increased animal survival.

**Conclusion:**

This study provides evidence that FUS-BBB opening can enhance immune-modulating agent delivery to the brain, which improve the anticancer immune response in brain tumor treatment.

## Introduction

Nearly 260,000 patients worldwide are diagnosed annually with primary malignant brain cancers, among which, more than 50% are reported to have glioblastoma multiforme (GBM) [1]. GBM is the most common malignant brain cancer in adults, and it is responsible for half of cancer patients’ deaths. The median survival times are reported to be 5–15 years for low-grade glioma patients, but only 9–12 months for high-grade glioma patients [2,3]. The current approach for brain tumor therapy is surgical resection with radiotherapy, which is typically accompanied by adjuvant and chemotherapy or other therapeutic molecule substance delivery into the tumor site [4,5]. Unfortunately, the therapeutic efficacy of most drugs is significantly limited due to the structure of the blood–brain barrier (BBB) or blood-tumor barrier, which limits the penetration of the therapeutic agents and ability to reach therapeutic dose at the target tumor site. The integrity of the BBB in the brain tumor is typically highly heterogeneous, resulting in highly variable BBB permeability within different tumor areas. The BBB is usually most permeable in the tumor core, whereas it remains relatively intact in the peripheral regions of the tumor [6]. The BBB of the peripheral glioma has been shown to remain highly functional [7-9], and previous clinical studies have demonstrated that brain tumor cells can migrate great distances from the enhancing regions of the tumors [10]. As a result, current therapeutic substance delivery into brain tumors faces several difficult challenges.

Passive brain-tumor immunotherapy is a brain-targeting delivery strategy that faces the same challenges of limited blood–brain permeability caused by the BBB. The concept of passive brain-tumor immunotherapy typically refers to the delivery of immune-effector cells and/or a variety of molecules including monoclonal antibodies and cytokines into brain tumors. The aim is to deliver cytokines or other immune-triggering substances at a sufficiently high concentration locally so that they can effectively trigger an antitumor immune response and establish long-term immunity against tumor recurrence in the host. There have been attempts to deliver interleukin-2 (IL-2) [11,12], interleukin-4 (IL-4) receptors [13,14], interleukin-12 (IL-12) [15], interleukin-13 (IL-13) receptor protein [16], transferring-diphtheria toxin [17], tumor growth factor-gamma, and tumor-specific cytotoxic T lymphocytes (CTLs) [18]. However, to overcome BBB blockage, most of the immune-triggering substance are delivered through local injection, making the procedure invasive. Among the above-mentioned immune-triggering substances, IL-12 is of particular interest due to its role in immunity and tumor angiogenesis. First, IL-12 has been reported to possess anti-angiogenic properties and have an anti-glioma effect because it can stimulate an antitumor immune response. Liu et al. reported the use of a replication-deficient adenoviral vector encoding IL-12 for treatment of a murine glioma model, and demonstrated that intratumoral delivery of gene-transfer IL-12 reduced tumor volume and prolonged survival in a GL-26 glioma model [19]. Using a continuous infusion system, Jean et al. demonstrated significant tumor regression using local intracranial cytokine delivery [20]. On the other hand, Salmaggi et al. analyzed the intracavitary level of vascular endothelial growth factor (VEGF) and IL-12 in 45 patients, and found that higher intracavitary concentration of VEGF and lower IL-12 corresponds to higher grade of glioma and shorter patient survival [21]. This demonstrates that there is a correlation between IL-12 level and brain tumor prognosis. Although systemic administration of recombinant IL-12 in a variety of rodent tumor models has demonstrated promise in significantly suppressing tumor growth and enhancing animal survival [22,23], the success is primarily limited because the BBB prevents the achievement of therapeutic levels in patients, thereby increasing the systemic concentration and the immune toxicity [24-26].

Focused ultrasound (FUS) exposure combined with IV-injected microbubbles has recently been shown to locally and temporally open the blood–brain barrier, thus providing a new opportunity for effective local drug delivery to brain tumors [27,28]. This BBB disruptive effect was found to be temporary and reversible without damaging surrounding central nervous system (CNS) tissues or neural cells [29]. The intravenous administration of microbubbles allows selective disruption of the BBB by significantly reducing exposure to ultrasonic energy and decreasing the influence on the parenchyma, thus minimizing the off-target effect [30]. Compared to other approaches such as local enhanced injections, or carotid infusions, FUS thus presents a competitive and attractive alternative for local induction of BBB disruption to increase the local concentrations of chemotherapeutic agents in GBM [31-33]. We therefore hypothesized that there was an opportunity to locally enhance IL-12 delivery deposited at a targeted tumor site via FUS-induced BBB opening to both improve brain tumor immunotherapy and anti-angiogenesis for glioma therapy.

**Figure 1:**
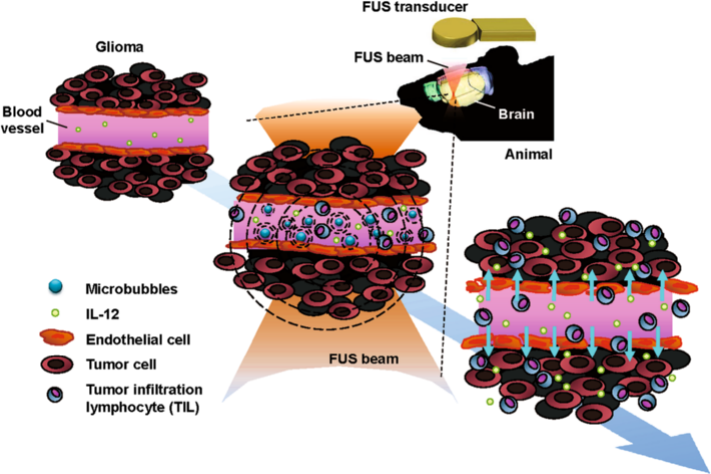
**Schematic of FUS-induced BBB opening to enhance IL-12 delivery in brain glioma treatment.**

The aim of this study was to apply focused ultrasound to temporally open the blood–brain barrier, and to evaluate the synergistic effect from concurrent delivery of IL-12 to improve the glioma-suppressing effect. Figure 1 summarizes the concept of synergetic FUS-induced BBB opening to enhance targeted IL-12 delivery. In this study, we aimed to verify that: (1) focused ultrasound can enhance the local permeability to allow penetration of the therapeutic molecules into the brain tumor, (2) the systemic administration of safety level IL-12 did not induce a systemic cytotoxic immune effect, and (3) combined FUS-induced BBB opening and safe IL-12 delivery can trigger local immunological effects to improve an anti-tumor effect.

## Materials and methods

### Glioma model

All animal experiments were approved by the animal committee (Chang-Gung University, Taoyuan, Taiwan) and adhered to the experimental animal care guidelines. Pathogen-free male Sprague–Dawley rats (200–225 g) were purchased from the National Laboratory Animal Center (Taipei, Taiwan). C6 glioma cells were harvested by means of trypsinization and cultured at a concentration of 1 × 10^5^ cells/mL for implantation. A total of 5 μL of C-6 glioma cell suspension were injected at a depth of 4.5 mm from the brain surface. The injection was performed over a 10-min period, and the needle was withdrawn over another 2 min.

**Figure 2:**
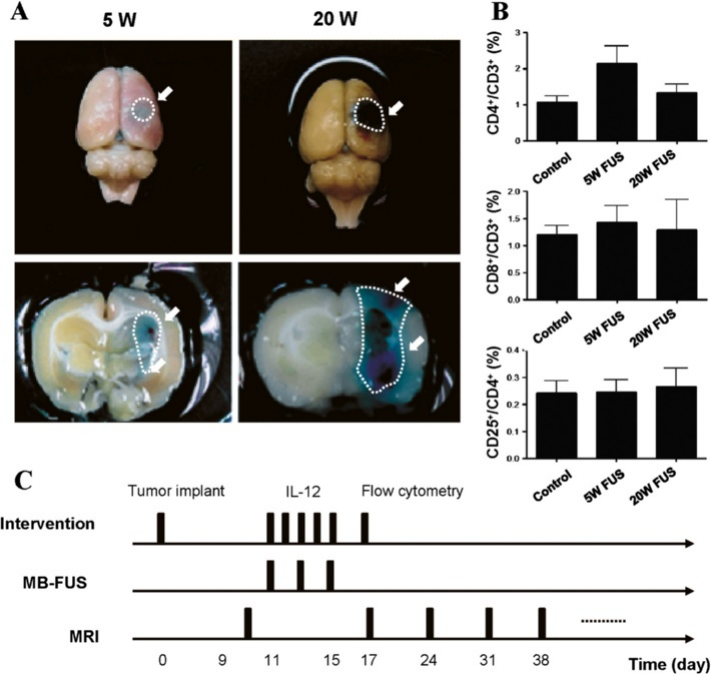
**FUS-BBB opening confirmation/ immune-response in normal rats and experimental timelines. (A)** Gross brain sections to assess Evans blue dye leakage in brain tissue exposed to 5-W FUS-induced BBB opening as well as the combined large-scaled erythrocyte extravasations after 20-W FUS exposure). **(B)** Population comparison of CD3+CD4+, CD3+CD8+, CD4+CD25+ T lymphocytes in controlled, 5-W FUS-exposed, and 20-W FUS-exposed normal animals. The CD3+CD4+, CD3+CD8+, and CD4+CD25+ cells specifically represent populations of helper T lymphocytes (Th), cytotoxic T lymphocytes (CTL), and regulatory T lymphocytes (Treg), respectively. (n = 7 per group). **(C)** The experimental timeline.

Control rats were injected with C6 glioma cells, but received sham ultrasound procedure with no energy. A second group of rats was subjected to focused ultrasound at the selected pressure level (5 W) at day 11, day 13, and day 15 after tumor implantation. A third group of rats received a single dose per day for 5 days of IL-12 (0.3 μg/kg/day) via intraperitoneal injection (IP) from day 11 to day 15 after they were injected with the tumor cells. A fourth group of rats received 5-days IL-12 (0.3 μg/kg/day) IP combined with 3 times of 5-w focused ultrasound on day 11, 13, and 15. There are 12 rats in each group for flow cytometry, at least 12 rats in each group for efficacy and magnetic resonance image (MRI) study, and 3 rats in each group for immunohistochemical (IHC) study. Ten days after implantation, tumor sizes were measured using 7 Tesla MRI scanner. Animals were assessed longitudinally by MRI at one-week intervals up to day 38 to determine tumor size. The animals were anesthetized with 2% isoflurane throughout the MRI imaging process, placed in an acrylic holder and positioned in the center of the magnet. Tumor size was quantified using T2-weighted images with the following parameters: TR/TE = 2500 ms/68 ms, matrix size = 176 × 256, FOV = 31 × 35 mm (resolution = 0.18 × 0.14 mm). The treatment and evaluation timelines are shown in Figure 2.

### Focused ultrasound exposure

Animals were anesthetized with a mixture of oxygen (with flow rate of 0.8 L/min) and 2% vaporized isoflurane using an anesthesia vaporizer. The top of the cranium was shaved with clippers, and a PE-10 catheter was inserted into the tail vein for injections. The animal was placed directly under an acrylic water tank with its head attached tightly to the thin-film, 4 × 4 cm^2^ window at the bottom of the tank. A focused ultrasound transducer (Sonic Concepts, Seattle, WA, USA; operating frequency = 0.5 MHz, active element diameter = 64 mm, radius curvature = 55 mm) driven by an arbitrary function generator (33220A, Agilent, Palo Alto, CA, USA) with a radio-frequency power amplifier (150A100B, Amplifier Research, Souderton, PA, USA) for RF signal amplification and a power meter (Model-4421, Bird, USA) for electrical power sensing was used. FUS exposure was 5 or 20 Watt (W) in electric power, equivalent to measured acoustic negative-peak pressures of 0.36 – 0.7 MPa. Before FUS exposure, a 0.1 mL/kg bolus of microbubbles (MBs) (Sonovue, Bracco Diagnostics Inc., Milan, Italy) mixed with 0.2 mL of saline were injected intravenously (IV), followed by flushing with 0.2 mL heparin. A single sonication of burst mode ultrasound was delivered to the animal (burst length = 100 ms, pulse repetitive frequency = 1 Hz, exposure time = 90 s).

### Detection of peripheral/tissue lymphocytes

The animals were sacrificed on the day16^th^ after tumor implantation. The organs (brain, mesenteric lymph nodes (MLN) and spleen) were removed to determine the effect of FUS combined with IL-12 treatment. The left brain was chopped into small pieces using a razor blade and 1 g of brain was incubated with 10 ml collagenase type IV (1 mg/ml; GIBCO, CA, USA) in PBS buffer on a shaker incubator at 100 rpm, 37°C for 30 min and washed with RPMI1640 medium. Cells were passed through nylon mesh, centrifuged at 1800 rpm for 3 minutes, and washed with RPMI1640 media. Cell pellets were re-suspended in 8 ml RPMI1640 media and layered over 4 ml Ficoll (Pharmacia, Peapack, NJ) in a 15-ml centrifuge tube. After centrifuging at 2000 rpm for 20 minutes with a deceleration speed set at 2, the single-cell suspension was separated from the Ficoll, and leukocytes were recovered from the inter-phase.

### Antibodies and flow cytometry

Anti-CD3-FITC, anti-CD4-APC, anti-CD8-PE, anti-CD11-APC, anti-CD25-FITC and anti-CD45-FITC antibodies were used for intracellular staining. TILs were washed twice with Hank’s balanced salt solution (HBSS), then fixed and permeabilized in Fix/Perm buffer according to the manufacturer’s instructions for 30 min. Cells were washed twice with permeabilization buffer and then incubated with appropriate antibodies at 4°C for 30 min in the dark. Unbound antibodies were removed by washing twice with permeabilization buffer. Flow cytometry analyses were performed on a three-color fluorescence FACS caliburcytometer using Cell Quest software (Becton-Dickinson, CA, USA).

### Histological examination

To confirm the FUS-induced local immune response, rats with tumors were sacrificed 2 hours after 5-W FUS exposure on day 10. Paraformaldehyde-fixed and paraffin-embedded tumors were used to prepare 10-μm thick sections for IHC analysis. CD8+ marker (Santa Cruz; sc-53063) was employed to specifically bind to CTLs (CD3+/CD8+ TILs). For Treg cells (CD4+/CD25+ TILs), instead of using CD25+ marker, FoxP3 marker (Biosussa; bs-0269R) was employed because it specifically binds to Treg cells. The adjacent sections were stained with hematoxylin-eosin (HE) to observe histological changes after FUS exposure.

### IL-12 concentration measurement

To determine IL-12 concentration in brain tumor tissue, four groups with control, FUS once, single dose of IL-12 IP and FUS+ IL-12 treatment were performed at day 11 after tumor implantation. There were eight tumor-bearing rats for each group. Animals were sacrificed 24 hrs after treatment, with tumor tissues were collected and homogenated to perform rat ELISA analysis (IL-12p70 kits, Invitrogen).

### Magnetic resonance imaging and analysis

Tumor-bearing rats were followed to monitor the progression of brain tumors. All MRI images were acquired on a 7-Tesla magnetic resonance scanner (Bruker ClinScan, Germany) and a 4-channel surface coil was used on the top of the rat brain. The animals were anesthetized through inhalation of 2% isoflurane throughout the MRI process, placed in an acrylic holder and positioned in the center of the magnet. In the tumor animal experiment group, tumor size was quantified using turbo-spin-echo based T2-weighted images with the following parameters: pulse repetition time (TR)/echo time (TE) = 2000/41 ms; FOV = 33 × 50 mm^2^ (162 × 320 pixels); slice thickness = 0.5 mm. The relative tumor size was estimated by measuring the single image slide containing the maximum tumor area, and animals were longitudinally imaged every 7 days for up to 38 days after the 1st MRI screening (i.e., day 10). Detailed experimental designs were shown in Figure 2(C).

### Statistical analysis and tumor volume measurement

Flow cytometry data are displayed as means ± standard deviations. The Mann–Whitney *U* test was used for the statistical analysis of differences between groups. Log-rank test was used for survival analysis. Calculations were performed with PRISM (GraphPad, version 5.0). Differences were considered statistically significant when *p* < 0.05 (labeled as *; further labeled as ** when *p* < 0.001).

## Results

### FUS has minimal effect on T cell components in normal rat brain

Figure 2(A) shows typical brain sections stained with Evans blue to mark the BBB-opened regions in normal rats. FUS was applied to normal rats and was targeted to the right striatum separately for 7 rats in each group. An exposure power level of 5 W induced a successful BBB opening effect, confirmed by Evans Blue staining in the exposed brain hemisphere. HE stains also confirmed that the brain tissues did not show any pathological changes (not shown). When a higher exposure level of 20 W was applied, the BBB-opened regions spread toward a wider area, with RBCs extravasated in the exposure regions (both confirmed by gross sections and HE stains).

The percentages of CD3+CD4+ lymphocytes, CD3+CD8+ lymphocytes and CD4+CD25+ lymphocytes, which represents helper T lymphocytes (Th), cytotoxic T lymphocytes (CTL), and regulatory T lymphocytes (Treg), respectively in normal rat brains for 3 groups (control, 5-W FUS exposure, and 20-W FUS exposure) are shown in Figure 2(B). Aside from a slight increase in Th cells (from 1.07 ± 0.47% to 2.14 ± 1.29% in 5 W exposure, but without statistically significance (*p* = 0.12), there were no changes in the populations of either CTL or Treg cells after FUS exposure. Overall, the T lymphocyte populations were not significantly influenced by FUS exposure either with an intact BBB opening or BBB-opening accompanied by RBC extravasations in normal animals. When considering both successful BBB-opening and safety with minimal possible tissue hazard induced by FUS exposure, a FUS exposure level of 5 W was selected and applied in subsequent animal experiments.

### FUS exposure enhances IL-12 influence on regional CD8+ T cell component, while having almost no effect on the systemic T cell component of brain tumor-bearing rats

Next, we designed an efficacy study to test whether FUS and IL-12 have a synergistic effect on brain tumor treatment and how this combined treatment influences both systemic and tumor regional T lymphocyte components. Tumor-bearing animals were sub-grouped as follows: (1) control, (2) FUS-induced BBB opening alone, (3) IL-12 delivery alone, and (4) combined FUS-induced BBB opening with IL-12 delivery.

**Figure 3:**
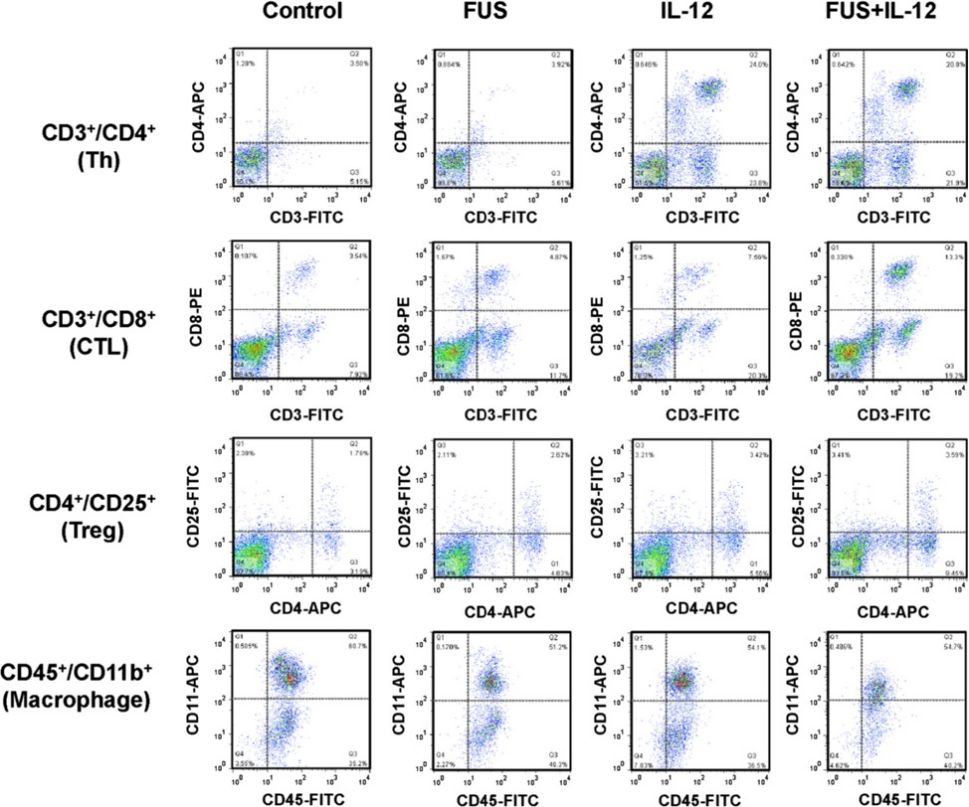
**Representative flowcytometric analysis.** Cell population comparison of CD3+CD4+, CD3+CD8+, CD4+CD25+ T lymphocytes and CD45+CD11b+ macrophages of tumor tissues obtained from glioma-bearing animals in control, FUS alone, IL-12 alone, and combined IL-12+FUS groups.

**Figure 4:**
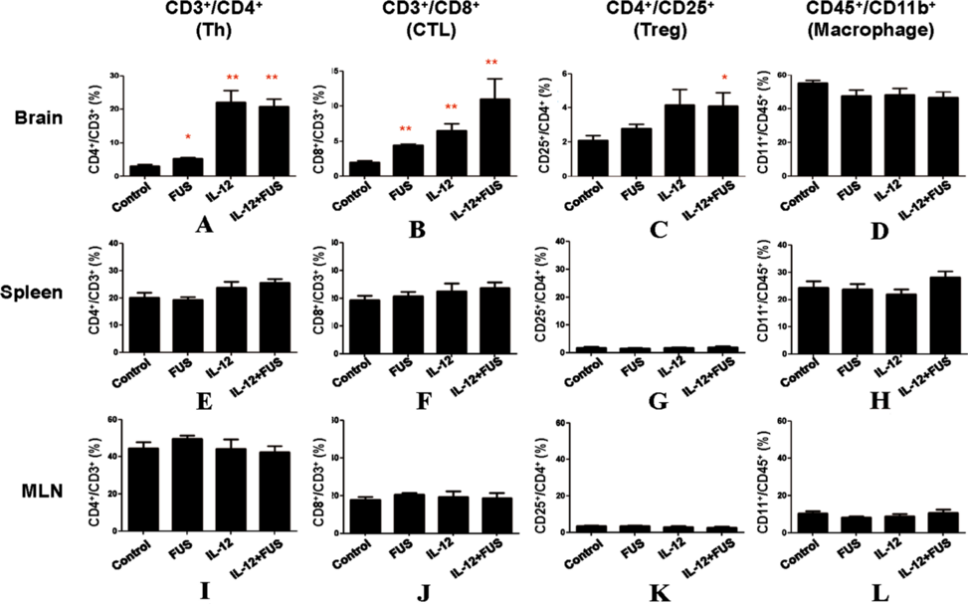
**T lymphocyte and macrophage populations.** CD3+/CD4+, CD3+/CD8+, CD4+/CD25+ T lymphocytes and CD45+/CD11b+ macrophages in glioma-bearing animals among the experimental groups (control, FUS-alone, IL-12 alone, and combined FUS/IL-12 groups). **(A-D)** In brain tumor; **(E-H)** In spleen; **(I-L)** In mesenteric lymph nodes (MLN) (n = 12 per group).

Figure 3 shows the typical results from flow cytometric analysis of the individual experimental groups with the sample obtained from the brain tumor tissues. The corresponding quantitated lymphocyte populations are shown in Figure 4. In the brain tumor region, the CD3+CD4+ TIL population showed no significant increase induced by FUS exposure alone when compared to sham group (*p* = 0.016), but was significantly increased by either IL-12 alone and combined FUS + IL-12 (22.16 ± 7.75% and 20.83 ± 5.28% with *p* = 0.002 and <0.001, respectively) (Figure 4(A)). CD3 + CD8+ lymphocytes were both increased significantly (both *p* = 0.004) either by FUS exposure alone (about 2 fold, from 1.99 ± 0.73% to 4.41 ± 0.58%) or by IL-12 administration alone (about 3 fold, from 1.99 ± 0.73% to 6.51 ± 2.01%), but with a most profound and increase in FUS + IL-12 group (about 5 fold, from 1.99 ± 0.73% to 10.97 ± 5.96%) (Figure 4(B)). The CD4+CD25+ lymphocyte population only significantly responded to the FUS + IL-12 group (about 2 fold, from 2.1 ± 0.74% to 4.11 ± 2.04%) (Figure 4(C)). There was no influence on the CD45 + CD11b macrophage population in the brain from the different treatments, indicating that neither FUS exposure, IL-12 administration, nor combined did not triggered macrophage-enhanced differentiation and invasion in the tumor region (Figure 4(D)).

**Figure 5:**
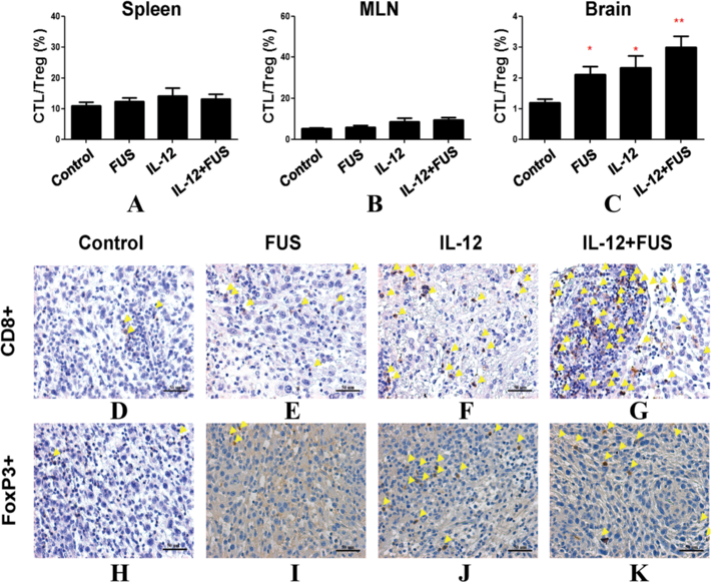
**T lymphocyte population ratio and IHC histological confirmation. (A-C)** T lymphocyte population ratio between CD3+/CD8+ (CTL) and CD4+/CD25+ (Treg) among different experimental groups in spleen **(A)**, in MLN **(B)**, and in brain tumor **(C)**. (n = 12 per group) **(D-K)** Immunohistological chemistry (IHC) staining to show specific T lymphocyte distribution among the experimental groups (**(D-G)**: Cytotoxic T lymphocytes; Regulatory T lymphocytes; n = 3 per group).

Compared to the change in the population of enhanced specific lymphocytes (particularly for CTLs or Treg) induced either by FUS, IL-12, or the combination FUS/IL-12, there were no significant changes in the lymphocyte population percentages systemically, either in spleen (Figures 4(E) and 5(H)) or in MLN (Figures 4(I) and 5(L)). This indicates that combining FUS with IL-12 administration only triggers the anticancer-specific immunological response in the targeted tumor regions.

Regulatory T lymphocyte has been reported to play an immune inhibitory role, and CD8 + T cells may act as effectors in the tumor microenvironment. We therefore examined the changes in the ratios of CD8 + T cells/Treg cells locally in brain tumors as well as systemically, as illustrated in Figure 5. There is no obvious CTL/Treg ratio change in MLN and spleen (Figure 5(A) and (B)). On the other hand, in glioma tissues, it was observed that both FUS-BBB opening and IL-12 indeed resulted in an increase in the CTL/Treg ratio when compared with the control group (Figure 5(C); control group: 1.19 ± 0.38, FUS group: 2.12 ± 0.70, *p* = 0.035; IL-12 alone group: 2.34 ± 0.91, *p* = 0.023). Combining FUS-BBB opening with IL-12 administration provided the most profound CTL/Treg ratio increase (increase to 3.0 ± 0.99, *p* < 0.001), indicating a synergistic effect on immunological changes in the tumor region that are beneficial in the suppression of glioma progression. This change in the TIL population ratio was confirmed by IHC staining (Figure 5(D) – (K); instead of using CD25+, Treg cells were stained by FoxP3 marker to more specifically bind to Treg cells), showing that FUS primarily induces an increase in CTL population in tumors, but IL-12 can both trigger CTL and Treg cell population increase in tumors. Combined FUS exposure with IL-12 delivery therefore produced a beneficial increase in the CTL to Treg population ratio.

### FUS combined with IL-12 treatment increases IL-12 brain deposition, inhibits brain tumor growth and improves survival rate of rodents

**Figure 6:**
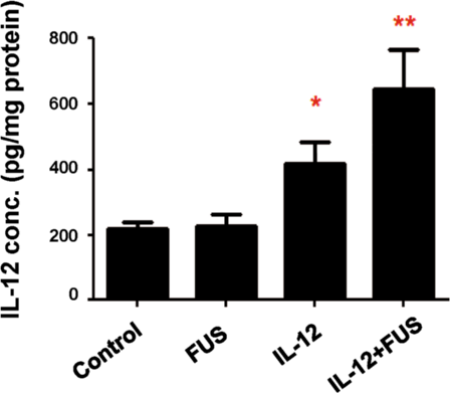
**Qantitated IL-12 concentrations deposited in brain tumors among experimental groups (n = 8 per group).**

Figure 6 shows the measured IL-12 concentration desisted at brain tumor site among each experimental groups. FUS exposure alone did not trigger IL-12 increase in tumor and the IL-12 level was close to the amount measured in control group (225.8 ± 98.4 versus 220.0 ± 61.8 pg/mg protein). IL-12 administrations alone significantly increased local IL-12 deposition in tumor about 1.89 fold (417.3 ± 168.5 pg/mg protein, *p* = 0.006). While combing FUS-induced BBB opening with IL-12 administration, The local IL-12 concentration at brain tumors can be further increased 2.87-fold when compared to control (632.1 ± 358.2 pg/mg protein; *p* = 0.0034).

**Figure 7:**
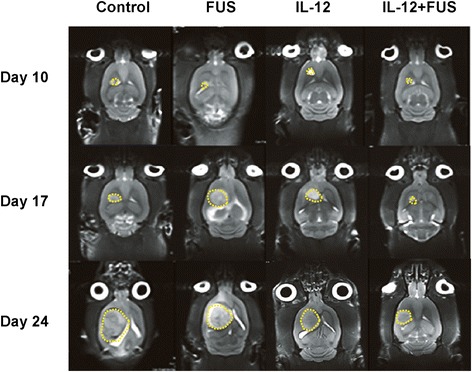
**Tumor progression followed by MRI.** Representative T2 MR imaging to follow brain tumor progression (7 days observation time interval; 3 time points in total) among the experimental groups.

**Figure 8:**
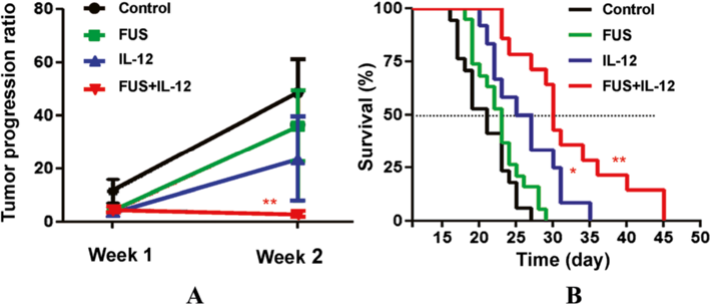
**Tumor progression and survival analysis. (A)** Corresponding tumor progression ratio in four animal groups. Week 1 = tumor progression ratio with time interval between the 1st and 2nd MRI; week 2 = tumor progression ration with time interval between the 2nd and 3rd MRI. (n = 6 per group) **(B)** Kaplan–Meier plot demonstrating animal survival among the experimental groups. (n = 12 for control and IL-12 groups, n = 13 for FUS and IL-12 + FUS groups).

To assess glioma treatment efficacy, we used MRI to longitudinally assess glioma progression from each experimental group. Figure 7 demonstrates typical T2 images used to quantitate tumor volume. The tumor progression ratio during the first week (days 10–17) and second week (days 17–24) were analyzed and presented in Figure 8(A). Glioma-bearing animals without any treatment showed fast tumor progression (from 11.41 ± 8.52 during week 1 to 48.82 ± 30.17 during week 2). FUS-induced BBB opening alone did not suppress tumor progression (from 11.41 ± 8.52 to 3.87 ± 4.81, *p* = 0.114 in week 1 and from 48.82 ± 30.17 to 35.95 ± 37.84, *p* = 0.345 in week 2 compared to control), whereas administration of IL-12 alone provided a moderate, but not statistically significant, suppression of tumor growth (from 11.41 ± 8.52 to 3.62 ± 1.22, *p* = 0.038 in week 1 and from 48.82 ± 30.17 to 23.62 ± 41.77, *p* = 0.101 in week 2 compared to control). Of note, we observed that combined FUS-induced BBB opening with IL-12 administration provided the most significant suppression of tumor progression when compared to control (from 11.41 ± 8.52 to 4.75 ± 3.23, *p* = 0.1714 in week 1 and from 48.82 ± 30.17 to 3.60 ± 3.77, *p* = 0.002 in week 2 compared to control).

The animal survival among the experimental groups is shown in Figure 8(B). FUS-BBB opening alone or IL-12 administration alone had less tumor growth inhibition and survival benefit when compared to control (median survival = 23 and 26 days, respectively, compared to 20 days or IST_median_ = 9.5% and 23.8%, respectively; *p* = 0.116 and 0.004). IL-12 treatment alone did inhibit tumor growth in the second week after treatment and improved survival of brain tumor-bearing rats. Of note, we observed that FUS-BBB opening combined with IL-12 administration produced the most powerful inhibition of tumor growth and survival benefit (median survival = 30 days, or IST_median_ = 42.9%; p <0.001). The detailed statistical results are shown in Table 1.

**Table 1:** **Efficacy of various treatment protocols for glioma-bearing animals among different experimental groups**

**Group (n)**	**Median survival (days)**	**IST**_**median**_ **(%)**	**Mean survival (days)**	**IST**_**mean**_ **(%)**	**Maximal survival (days)**	***p-value***
Control (12)	21	—	21.5	—	28	—
FUS (13)	23	9.5	23.9	11.2	32	0.116
IL-12 (12)	26	23.8	25.5	18.6	35	0.004
IL-12 + FUS (13)	30	42.9	31.2	45.1	45	<0.001

Increase in median survival time (IST_median_; in %), mean survival time (IST_mean_; in %) and statistical analysis (Log-rank test and *p*-value) are all relative to the control group (n = number of animals per group).

## Discussion

### Significance of this study

In this study, we demonstrated the synergetic effect of FUS-induced BBB opening combined with delivery of IL-12 to enhance the therapeutic effect of anti-glioma treatment in a preclinical small-animal study. We showed that FUS-induced BBB opening did not trigger significant TIL distribution changes, but did increase the total TIL numbers. While IL-12 administration significantly increased both distribution and population percentages of TILs, it did not contribute to end-point improvement as measured by tumor progression control and survival. When FUS-induced BBB opening was combined with IL-12 administration, the enhanced local delivery of IL-12 into glioma with the aid of transient opening of BBB successfully changed the treatment outcome both in terms of tumor progression control (from 2.4-fold to 13.5-fold of 7-day tumor progression) and animal survival (42.9% of improve). To our knowledge, this is the first report of FUS-induced BBB opening as a tool for facilitating anticancer immunotherapy against brain tumors. This study provides important information for combining non-invasive focused ultrasound with therapeutics to locally trigger an immune response for CNS disease treatment.

### Brain tumor immunotherapy

Enhanced delivery of IL-12 for immunotherapy of glioma has been attempted previously in both preclinical and clinical studies. For preclinical evaluation, Kishima et al. and Kikuchi et al. showed that survival can be improved when IL-12 is systemically delivered in a preclinical murine model [34,35]. Jean et al. demonstrated that combined systemic IL-12 delivery with irradiation to tumor cells can synergistically improved immunological suppression of 9 L glioma progression in an animal study [20]. On the other hand, DiMeco et al. used genetically engineered 9 L glioma cells to express IL-12 as a source of locally delivered cytokine IL-12 and also showed improvement in animal survival [15]. Also, Liu et al. demonstrated that delivery of IL-12 in a glioma animal treatment model had a similar tumor-suppressing effect and also found the anti-tumor immunity was triggered by increased TIL infiltration, including CD4+ and CD8+ T lymphocytes [36,37], which is similar to the observation in this study. In terms of clinical studies, Ren et al. used convection-enhanced delivery to enhance IL-12-expressing viral vectors in a phase I/II study, and confirmed the safety of the approach and ability to locally enhance IL-12 levels at the brain tumor site [38]. Kichuchi et al. [39] also investigated the safety of combined infusions of dendritic and glioma cells with recombinant IL-12 for the treatment of malignant glioma in humans. In their study, GBM patients showed significant reduction (>50% in tumor mass reduction) of tumor burden, providing evidence of the glioma-suppressing effect of IL-12.

### Mechanism of IL-12 in anticancer response

We demonstrated that FUS-induced BBB opening can enhance IL-12 penetration and deposition at brain tumors (shown in Figure 6) and therefore improve brain tumor immunotherapy (Figure 8). The roles and mechanisms of IL-12 as an immunological antitumor agent have been exploited extensively since its discovery in the 1990s. IL-12 is physically secreted at the antigen site by immune cells such as macrophages, B-cells, and microglia. Also, in addition to being an important element in the immune system, IL-12 is a potentially powerful antitumor cytokine [40,41]. It has been shown that the presence of IL-12 can enhance proliferation of T cells [42,43], and also facilitate interferon (IFN)-gamma production to promote Th-1-mediatedantitumor cytotoxic immunity [44] (Th1, one of the CD4+ helper T cells plays important roles in the enhancement of immunity) and the associated anti-cancer immunological response (IL-12 were reported to facilitate the development of IFN-gamma-secreting tumor-specific Th1 T cells and TILs, thereby enhancing the tumor-killing effects) [45]. In addition to the role of IL-12 in anticancer immunological responses, IL-12 can also regulate angiogenesis and serve as an anti-angiogenic factor against tumor progression [46,47].

### Micro-environment changes caused by FUS-induced BBB opening

Besides of direct IL-12 deposition to trigger brain cancer immunotherapy, it is well known that FUS exposure in the presence of microbubbles can increase vascular permeability. These capillary permeability changes in various organs and tumor tissues possibly changes the tumor micro-environment which is beneficial for triggering anti-cancer immunity [48-51]. A previous study by Miller et al., in the absence of immunological observations, demonstrated a profound tumor suppression effect within 4 days caused by FUS exposure in the presence of microbubbles [52]. In this study, we confirmed that FUS exposure combined with microbubbles can transiently open the BBB, and we hypothesized that it provides transient micro-vascular and micro-environmental changes in the tumor bed, leading to an increase in tumor cytokine/chemokine release and triggering TIL infiltration. We also observed that simply changes in the micro-environment did not enhance systemic or local immunological response in normal tissue, but did significantly induce the infiltration of CTLs into tumors and also increased the ratio of CTL/Treg, which is a significant index of positive anticancer immune-triggering activity similar to that reported in previous clinical studies [53,54].

## Conclusion

In this study, we demonstrated enhanced local IL-12 delivery into glioma with the aid of FUS-induced transient opening of BBB, which improved TIL infiltration, triggered anticancer immunological response, and improved glioma treatment efficacy. This study provides useful information regarding the use of FUS-induced BBB opening to assist immune-modulating agent-enhanced delivery to benefit anticancer immune response for brain tumor treatment.
